# Improvement of the BALB/c-3T3 cell transformation assay: a tool for investigating cancer mechanisms and therapies

**DOI:** 10.1038/srep32966

**Published:** 2016-09-09

**Authors:** Doerte Poburski, René Thierbach

**Affiliations:** 1 Institute of Nutrition, Friedrich Schiller University Jena, Dornburgerstraße 24, 07743 Jena, Germany

## Abstract

The identification of cancer preventive or therapeutic substances as well as carcinogenic risk assessment of chemicals is nowadays mostly dependent on animal studies. *In vitro* cell transformation assays mimic different stages of the *in vivo* neoplastic process and represent an excellent alternative to study carcinogenesis and therapeutic options. In the BALB/c-3T3 two-stage transformation assay cells are chemically transformed by treatment with MCA and TPA, along with the final Giemsa staining of morphological aberrant foci. In addition to the standard method we can show, that it is possible to apply other chemicals in parallel to identify potential preventive or therapeutic substances during the transformation process. Furthermore, we successfully combined the BALB/c cell transformation assay with several endpoint applications for protein analysis (immunoblot, subcellular fractionation and immunofluorescence) or energy parameter measurements (glucose and oxygen consumption) to elucidate cancer mechanisms in more detail. In our opinion the BALB/c cell transformation assay proves to be an excellent model to investigate alterations in key proteins or energy parameters during the different stages of transformation as well as therapeutic substances and their mode of action.

Cancer is a leading cause of death worldwide and the number of new cases is expected to rise about 70% over the next two decades. More than 30% of cancer can be prevented by avoiding risk factors and others can be detected early or treated accurately^1^. Drug development for cancer therapy is time consuming and cost-intensive and most of the compounds fail the initial research phases in human^2^,^3^. To minimize the number of promising compounds analyzed in numerous and long-lasting animal studies it is important to better understand the mode of action and prospects of suitable drug candidates. Hence, we need a basic technology that allows us to screen for new therapeutic substances and ideally to study their mode of action.

Malignant cell transformation *in vitro* is described as a progressive process through qualitatively different stages^4^ and the involved cellular and molecular events are similar to those of *in vivo* multistage carcinogenesis^5^. The phenomenon of cell transformation involves phenotypic alterations (e.g. spindle-shaped morphology; basophilic staining), changes in growth behavior and control (e.g. immortality, multi-layered and acquisition of anchorage independent growth) as well as tumorigenicity when applied in susceptible animals^2^,^5^,^6^,^7^,^8^. After chemical treatment, these assays monitor the induction of malignant features in mammalian cells and their transition from normal to transformed cells^2^,^8^. In the last years a great effort was made to develop and validate alternative methods like the *in vitro* cell transformation assays (CTAs) to avoid unnecessary carcinogenicity testing with animals^9^. Although CTAs don’t simulate the whole *in vivo* neoplastic process, they can provide essential information regarding the identification of potential carcinogens and there mode of action^10^. Furthermore, they are faster, less expensive than the 2-year rodent bioassays and to date the only well-established method with the potential to detect both genotoxic and non-genotoxic carcinogens^8^,^11^.

There are two main CTAs used: the Syrian hamster embryo cell (SHE) assay developed by Berwald and Sachs^7^ and the BALB/c-3T3 cell transformation assay (BALB-CTA) according to Kakunaga^12^. The SHE assay is designed of target cells onto a feeder layer, which are treated with chemical agents 24 hours after seeding up to 7 days^5^. This method is intended to detect early stages of carcinogenicity and leads to morphologically transformed colonies^13^. Several modifications of the classical method had been carried out, like the use of medium with pH 6.7^14^,^15^ or an initiation-promotion protocol^16^.

The BALB-CTA is based on the immortalized embryonic mouse fibroblasts BALB/c-3T3^17^ using the subclone A31-1-1 by Kakunaga and Crow^18^. BALB/c-3T3 cells form normally a monolayer culture and get contact-inhibited after reaching confluence. Upon treatment with chemical agents, some cells do not stop proliferation and grow as morphologically aberrant foci over the monolayer of normal cells^2^,^6^. The original procedure consists of a 3 day exposure time to chemicals, 24 hours after seeding^12^,^19^. Cultures are further maintained 4 to 6 weeks with two medium changes a week until fixation with methanol. Morphologically transformed foci can be visualized by basophilic staining with Giemsa and therefore classified in three different types of foci^9^. Different improvements of the standard protocol were proposed, like a two-stage assay with treatment of suspected carcinogens followed by a known tumor promotor^20^, the use of the new developed Bhas 42 cell line (BALB/c-3T3 transfected with v-Ha-ras)^21^,^22^,^23^ or the combination of the BALB-CTA with microarray-based toxicogenomics^24^.

Despite the identification of potential tumor initiators and promotors by using cell transformation assays as standard toxicological methods we further improved the BALB-CTA for mechanistic cancer research. Here we present, that the classical two-stage model of the BALB-CTA can be combined with a parallel treatment of interesting substances to drive cell colony formation up or down. In addition, we successfully expanded the BALB-CTA for several endpoint applications, like analysis of protein level and signaling (westernblot, immunofluorescence, subcellular fractionation) as well as parameters of energy metabolism (glucose and oxygen consumption). Thereby, the BALB-CTA is most suitable for providing essential information regarding key proteins and their signalling during the different stages of transformation and to identify potential cancer therapeutics.

## Results and Discussion

**Figure 1:**
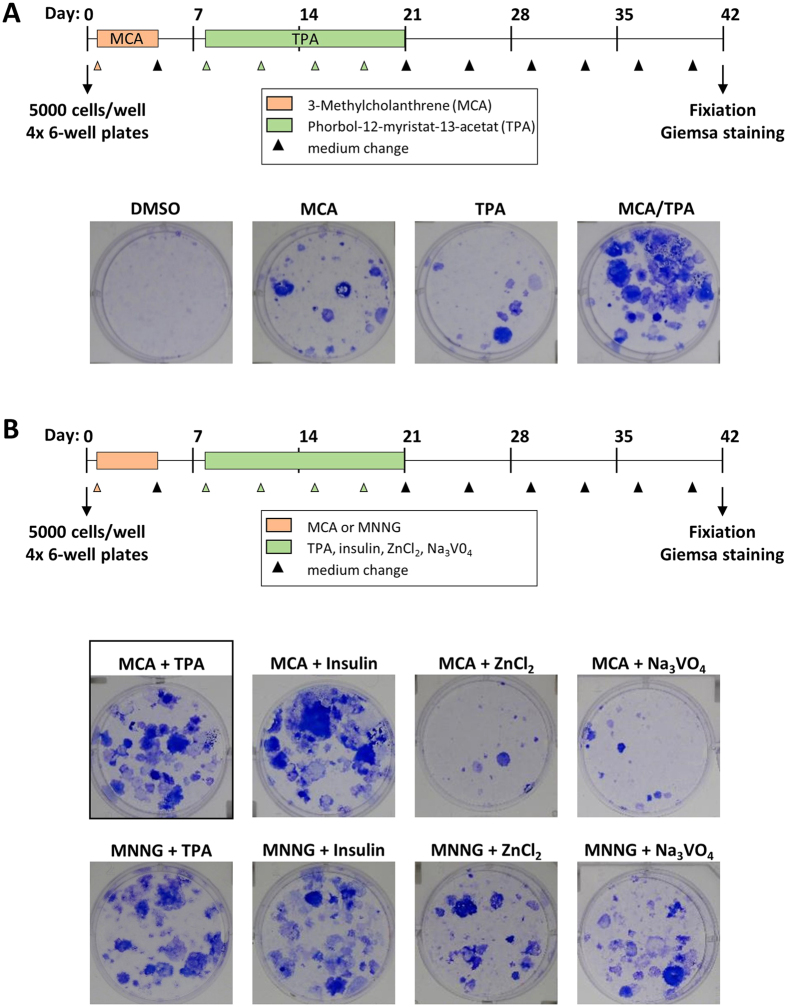
Standard two-stage BALB-CTA. (**A**) In the standard protocol of the BALB/c-3T3-cell transformation assay cells are treated with MCA as tumor initiator (day 1–4) and TPA as tumor promotor (day 8–21). After 42 days cells were fixed with methanol and malignant foci are stained with Giemsa (blue colored). (**B**) Other chemicals than MCA/TPA (framed) can be used as tumor initiators (MNNG) and tumor promotors (Insulin, ZnCl_2_ and Na_3_VO_4_) in the two-stage model of the BALB-CTA, but they differ in their colony formation efficiency.

**Figure 2:**
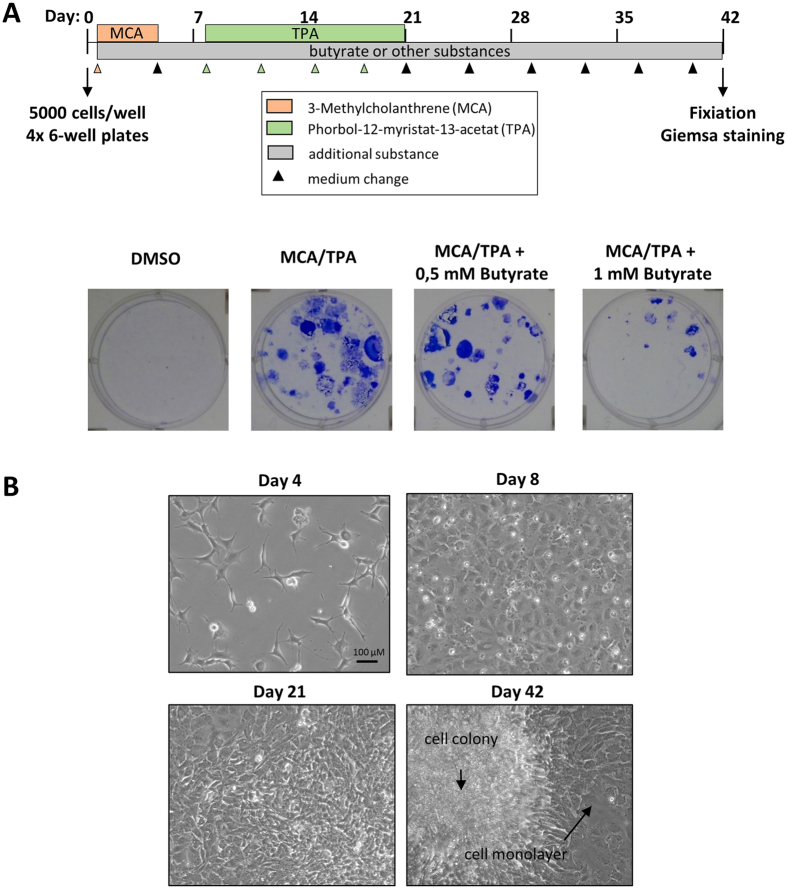
Improvement of the standard BALB-CTA. (**A**) An additional treatment with potential therapeutic substances (shown as butyrate as an example) is possible throughout the whole transformation assay (day 1 to 42) and leads to a different outcome of cell foci formation (blue colored by Giemsa staining). (**B**) Pictures illustrate the morphological changes of BALB/c cells at different days of the transformation protocol. Cells are seeded in at a low density and get contact-inhibited after reaching confluence. If cells are transformed by MCA/TPA treatment they start to pile up and grow over the monolayer of normal cells (day 21 or 42).

The BALB-CTA mimics some stages of *in vivo* carcinogenicity and is designed to evaluate the formation of morphological aberrant foci by several chemicals. In the standard two-stage model (Fig. 1A) cells are treated with a known tumor initiator for 72 h one day after seeding. After 4 days with normal medium the additional treatment with a known tumor promotor from day 8 to 20 leads to transformation of cells, which start to grow over the normal contact-inhibited monolayer. Until day 42 the cells receive normal medium twice a week and are finally fixed with methanol. The morphological aberrant foci can be visualized by basophilic staining with Giemsa and if required, can be classified into three different types of foci^9^. Reproducibility and efficiency of the BALB-CTA protocol was shown by the effects of well-known carcinogens. DMSO is used in the standard BALB-CTA as the solvent negative control and leads to no Giemsa stained foci at all (Fig. 1A). The separate usage of the tumor initiator MCA or tumor promotor TPA leads as expected to a small amount of transformed cell foci^6^,^25^. Nevertheless, only the combination of MCA treatment followed by TPA is highly efficient in cell transformation and leads to an enormous multilayer growth, which appears dark blue after Giemsa staining^25^,^26^. The usage of other known tumor initiators like MNNG^27^ and promotors like insulin, zinc chloride and sodium orthovanadate^28^ are also possible with this method (Fig. 1B) and only differ in the amount of arising cell colonies. Another possibility of the BALB-CTA is the additional treatment to MCA/TPA with a substance of interest for cancer prevention or therapy, which we demonstrated as an example with the short-chain fatty acid butyrate. Consistently to the described chemopreventive properties of butyrate in the literature^29^,^30^, a treatment with butyrate during the whole assay (day 1 to 42) leads to a concentration dependent decrease in cell transformation (Fig. 2A) and a clearly morphological change of the cells (Fig. 2B). These findings impressively show, that the BALB-CTA is not limited to evaluate potential carcinogens as a standard toxicological method, but can be also helpful to find and characterize cancer prevention and therapy candidates.

To further evaluate the underlying mechanisms of decreased cell transformation, a first step was to transfer the BALB/c two-stage model from the classical 6-well plate into a wide range of plate and dish formats (Supplementary Fig. S1). The 96-well plate is quite useful to test several substances in a high-throughput manner, whereas the use of a 10-cm dish is needed to achieve high amounts of protein or cells. For fluorescence analysis and high-end microscopy the different μ-slides form ibidi proved to be quite beneficial. Adequate protocol performance for other cell culture formats was verified by treatment with MCA and TPA with additional Giemsa staining.

**Figure 3:**
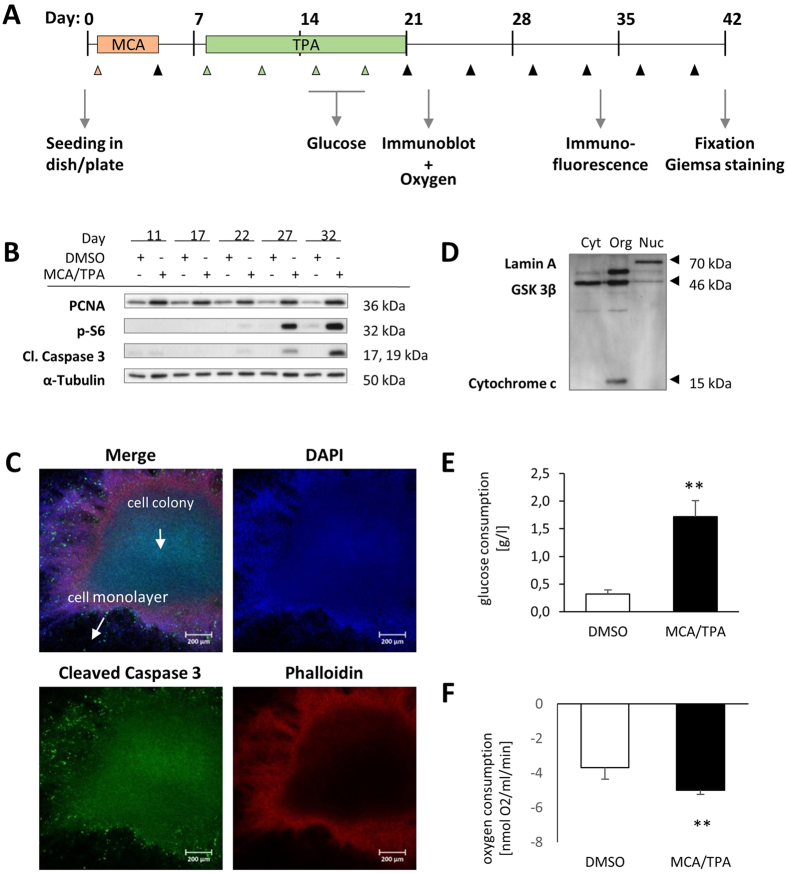
Combination of the BALB-CTA with several endpoint applications. (**A**) Illustration of the performed endpoint methods during the whole transformation protocol. Endpoint measurements can be realized variable at any day of the CTA. (**B**) BALB/c cells were treated according to the standard assay protocol with DMSO or MCA/TPA in a 10-cm dish for 11, 17, 22, 27 or 32 days. After preparation of protein samples immunoblot analysis for PCNA, p-S6 and cleaved Caspase 3 were accomplished with α-Tubulin as loading control. (**C**) BALB/c cells treated with MCA and TPA after 33 days of transformation assay were fixed and analyzed by confocal immunofluorescent microscopy. Colors appear as blue for DAPI staining, green for cleaved Caspase 3 antibody detection and red for Alexa Fluor® 647 Phalloidin labeled actin filaments. (**D**) Subcellular fractionation of untreated BALB/c cells was achieved with different buffers and centrifugation steps. Enriched fractions of cytosolic (Cyt), organellar (Org) and nuclear (Nuc) proteins were isolated and verified by immunoblot analysis for Lamin A (nucleus), GSK3β (cytosol) and Cytochrome c (organelles). (**E**) Samples of the applied medium were collected at day 15 (basic value) and day 18 (terminal value) of the BALB/c cell transformation assay. Glucose concentration of the medium samples were measured to calculate the glucose consumption. Results indicated are mean + SD (n = 3). Statistical differences are displayed as **(p < 0.01) according to a two sample Student’s t-test. (**F**) Oxygen consumption was measured at day 22 of the transformation protocol using a Clark-type electrode. 2 Mio cells/ml were applied and the change of oxygen consumption monitored over 5 min. Results indicated are mean + SD (n = 4) and statistical differences are calculated as **(p < 0.01) according to a two sample Student’s t-test.

Only the successful adaption of the BALB-CTA into other culture systems made it possible to combine the assay with several endpoint applications and meet their special requirements. All these methods can be performed at any time point of the whole transformation protocol (Fig. 3A) and this makes it possible to understand the multistep process of cell transformation in more detail. For mechanistic research it is quite important to look at changes in protein expression and activation, to identify responsible key proteins during the cell transformation process. A BALB-CTA with control and MCA/TPA treated cells was performed and combined with a time-dependent immunoblot analysis of selected proteins (Fig. 3B). The known marker for proliferation PCNA (Proliferating cell nuclear antigen) showed for all time points of the transformation assay a basal expression in the control cells and an increase with MCA/TPA treatment. Proliferation and PCNA activation is stimulated at day 11 and 17 by TPA treatment probably by activation of protein kinase C^31^,^32^. Further PCNA expression after the cell promotion phase is still higher, which is due to the increasing proliferation of the transformed cells in the foci. We also detected in the MCA/TPA treated cells an increase in the phosphorylation of the S6 protein and the cleavage of caspase 3 over time. The activation of the mTOR/p70S6 kinase signaling pathway has been found in a lot of different human cancer types and is a target for treatment strategies^33^,^34^. The elevated apoptosis can be compared to cellular death seen in the inner core of solid tumors or similar *in vitro* models due to insufficient oxygen or nutrient supply^35^.

With immunoblot analysis we can get a first idea which proteins can be involved when cells are treated with MCA/TPA or other substances, but we cannot differentiate between the signals of the transformed cell foci itself and the surrounding monolayer of normal cells. Therefore, to further investigate the precise location and activation of different proteins, we established the analysis by confocal immunofluorescence with up to three dyes. This method can be performed at any time of the transformation process, but adequate colony formation is beneficial. Thus, it was possible to ascribe the cleaved caspase 3 signal at day 33 to the developed cell foci itself and was not detectable in the surrounding monolayer of normal cells (Fig. 3C). These findings confirmed our results obtained by immunoblot analysis and made them even more comprehensible. Afterwards it is still possible to stain the existing foci with Giemsa for further characterization. An alternative method to analyze spatiotemporal protein changes, is the application of subcellular fractionation. We applied a fractionation protocol by Holden and Horten^36^ to our BALB/c cells and can differentiate between enriched fractions of the cytosol, organelles and nucleus. Successful fractionation was tested by performing immunoblots with typical proteins of the enriched fractions, like GSK 3β for cytosolic, cytochrome c for organellar and Lamin A for the nuclear fraction (Fig. 3D). This method makes it possible to detect alterations in the subcellular location of target proteins, for example transcription factors which translocate from the cytosol to the nucleus to hit their targets. Subcellular fractionation can be arranged, as all the other endpoint methods, at any day of the transformation protocol and can help to identify changes in initiation (day 1–4), promotion (day 8–21) or post-promotion phase (day 21–42) of the transformation assay.

From epidemiological studies and observations in human tumor types we know, that changes in energy metabolism can contribute to malignant cell transformation^37^. Therefore, we combined the BALB-CTA with measurements of energy metabolism, like glucose and oxygen consumption. In an early stage of transformation we detected an increase in glucose as well as oxygen consumption in the MCA/TP treated cells, which can be explained with the massive proliferation of the transformed cells (Fig. 3E,F). These methods in combination with the BALB-CTA gives us the possibility to monitor alterations in energy consumption at any day of the transformation process and learn more about the mode of action of preventive or harmful substances.

The BALB-CTA proves to be more than a toxicological method for chemical risk assessment to evaluate the initiating and promoting properties of chemicals. The successful transfer into other cell culture systems and combination with endpoint methods for protein analysis or energy parameter measurements make the BALB-CTA quite useful for mechanistic cancer research. This cheap alternative to rodent bioassays can provide valuable information of alterations of key proteins or cell metabolism during the whole transformation process in future. Moreover, the two-stage BALB-CTA can be combined with the additional treatment of preventive or therapeutic substances and can be helpful to investigate new treatment strategies.

## Materials and Methods

### Cell transformation protocol (CTA)

BALB/c-3T3-A31-1-1 (Hatano Research Institute, Japan) cells were cultured in DMEM/HAM’s F-12 (Biochrom #T481-10) containing 3 g/l D-glucose, 5% fetal bovine serum and 1% penicillin/streptomycin, to avoid unnecessary condition changes prior to the experiment. Cells were routinely maintained in a humidified incubator (37 °C, 5% CO_2_, 95% humidity) and only sub-confluent cells (about 70% confluence) were used for the CTA. The two-stage approach of the *in vitro* cell transformation assay with an initiation and promotion phase, is comparable to *in vivo* experiments^38^,^39^. The BALB/c 3T3 cell transformation assay was performed with some modifications to the recommended protocol of the EVCAM prevalidation study^40^. In order to create the same conditions during the whole experiment for further analysis in the initiation (day 1–4), promotion (day 8–21) or post-promotion phase (day 21–42), only DMEM/HAM’s F-12 medium was used, which proved to be very effective concerning the foci forming potential^41^. The duration of the assay was set to 42 days, to obtain sufficient colony formation. In our standard assay 5000 cells per well were seeded into 4 replicates of Corning® Primaria™ 6-well plates (VWR #734-0077) and cultured under standard conditions (37 °C, 5% CO_2_, 95% humidity) for 42 days. Medium changes took place every third or fourth day (see pattern of treatment), with additional treatment of 0.5 μg/ml MCA (3-Methylcholanthrene, Sigma #213942) on day 1 to 4 and 0,3 μg/ml TPA (12-O-Tetradecanoyl-phorbol-13-acetat, Sigma #79346) on day 8, 11, 15, 18 until day 21. Other substances of interest can be applied either completely (day 1–42) or just in the initiation (day 1–4), promotion (day 8–21) or even post-promotion phase (day 21–42) to analyze their effect on colony formation. After 42 days cells were washed twice with PBS, fixed with PBS/methanol (50:50) for 3 min and 100% ice-cold methanol for 10 min and washed twice with methanol. For analyzing the tumor forming potential cells were further stained with Giemsa (AppliChem #A0885) and transformed cell foci appear blue colored. Giemsa staining was achieved as followed: 3 min Giemsa solution (1 ml/well), adding deionized water (3 ml/well) for further 3 min, 5 times washing with tab water followed by 5 × 10 min washing with deionized water on the shaker. Other substances applied in the BALB/c assay were 1 μg/ml MNNG according to Ao *et al*.^27^, 20 μg/ml Insulin, 7.5 μg/ml ZnCl_2_ and 1 μg/ml Na_3_VO_4_ on the basis of Maeshima *et al*.^28^. Butyrate concentrations of 5, 10 or 20 mM were not tolerated by the BALB/c cells (data not shown), why 1 mM and below was chosen as concentrations for the CTA.

### Protein extraction and immunodetection

Protein samples were prepared by lysing (Cell signaling lysis buffer) and sonicating (Bandelin Sonopuls, Berlin, Germany) of the cells and quantified according to Bradford’s method^42^. SDS-PAGE was performed with a 10% gel and 30 μg protein extract per lane. The separated proteins were transferred to a PVDF membrane by semi-dry western blotting, followed by incubation with different antibodies. Antibodies used were PCNA (Cell Signaling #2586), p-S6 (Cell Signaling #2211), cleaved Caspase 3 (Cell Signaling #9664), anti-rabbit (Cell Signaling #7074) and α-Tubulin (Sigma T9026).

### Detection of glucose in culture medium

Cells were treated according to the standard cell transformation protocol in a 6-well plate. On day 11 medium was changed to DMEM/HAM’s F-12 without phenol red in preparation of the glucose determination (3 g/l glucose) and cells were further maintained without phenol red for the rest of the experiment. After medium change on day 15 medium samples for each well were collected. Cells were maintained for further 3 days (until day 18) and another sample of the consumed medium was collected. Glucose concentrations of the medium samples (without phenol red) were analyzed with the Glucose Assay Kit by Sigma (#GAGO20) following the manual instructions. Glucose consumption after 3 days of incubation was calculated.

### Oxygen consumption

Oxygen consumption was measured by using a Clark-type electrode (Hansatech Instruments; Norfolk, UK). Therefore cells were washed, trypsinized and counted with a Neubauer chamber. A solution of 2 Mio cells was filled into the air-tight and 37 °C tempered Clark electrode chamber to monitor the respiration rate for 5 min. Available Oxygen in the chamber passes through the teflon membrane to reduce the platinum cathode, meanwhile the silver anode is oxidized. The produced current by the electron shifting was used to calculate the respiration rate.

### Subcellular fractionation

Fractionation of cultured cells in 6-well plate format was done according to a protocol by Holden and Horten^36^. Fractions enriched for cytosolic proteins (digitonin-buffer: 150 mM NaCl; 50 mM HEPES; pH 7.4; 25 μg/ml digitonin), membrane bound organellar proteins (IGEPAL-buffer: 150 mM NaCl; 50 mM HEPES; pH 7.4; 1% IGEPAL) and nuclear proteins (RIPA-buffer: 150 mM NaCl; 50 mM HEPES; pH 7.4; 0.5% sodium desoxycholate; 0.1% SDS; 1 U/ml benzonase) were isolated and quantified by Bradford’s method^42^. Successful fractionation can be tested by performing immunoblots with 5 μg protein per lane and antibodies against GSK3-β for cytosol proteins (Cell Signaling #9315), Cytochrome c for organelles (BD Bioscience #556433) and Lamin A for nuclear proteins (Sigma L1293).

### Confocal immunofluorescence microscopy

Cells were treated according to the standard cell transformation protocol in a μ-slide 8-well (ibidi #80826). After 33 days cells were washed with PBS and fixed for 20 min with 2% para-formaldehyde and 10 min with 0.1% Triton® X-100. After washing with 1% bovine serum albumin as blocking solution (BSA) cell nuclei were stained with DAPI (Sigma D9542) and actin filaments with Alexa Fluor® 647 Phalloidin (Cell Signaling #8940). Afterwards the primary antibody cleaved Caspase 3 (Cell Signaling #9664) was applied, followed by the secondary antibody anti-rabbit (Cell Signaling #4412). Washing 2× with BSA was done between each incubation. Finally cells were covered with ibidi mounting medium (ibidi #50001) and analyzed with a Zeiss LSM 780 confocal microscope. After immunofluorescence analysis mounting medium can be washed away with 3× PBS and cells can be further stained with Giemsa.

### Statistical analysis

Calculations of statistical differences of glucose and oxygen consumption measurements were assessed according to a two sample Student’s t-test (equal variances). A probability value of p < 0.05 was considered to be statistically significant.

## Additional Information

**How to cite this article**: Poburski, D. and Thierbach, R. Improvement of the BALB/c-3T3 cell transformation assay: a tool for investigating cancer mechanisms and therapies. *Sci. Rep.* **6**, 32966; doi: 10.1038/srep32966 (2016).

## Supplementary Material

Supplementary Information
