# Supplementary material for: Improvement of the BALB/c-3T3 cell transformation assay: a tool for investigating cancer mechanisms and therapies
Source: Sci Rep. 2016 Sep 9;6:32966. doi: 10.1038/srep32966 (PMC5017208; doi:10.1038/srep32966)
Supplement: Supplementary Information [file srep32966-s1.doc]

**Titel:** Improvement of the BALB/c-3T3 cell transformation assay: a tool for investigating cancer mechanisms and therapies

**Authors:** Doerte Poburski^1^, René Thierbach^1^

^1^ Institute of Nutrition, Friedrich Schiller University Jena, Dornburgerstraße 24, 07743 Jena, Germany

**Corresponding Author:**

Dr. René Thierbach

Department of Human Nutrition

Institute of Nutrition

Friedrich Schiller University Jena

Dornburgerstraße 24, 07743 Jena

Phone Number: 0049/3641-949634

Email: [rene.thierbach@uni-jena.de](mailto:rene.thierbach@uni-jena.de)

**Supplement Figure S1:**

**
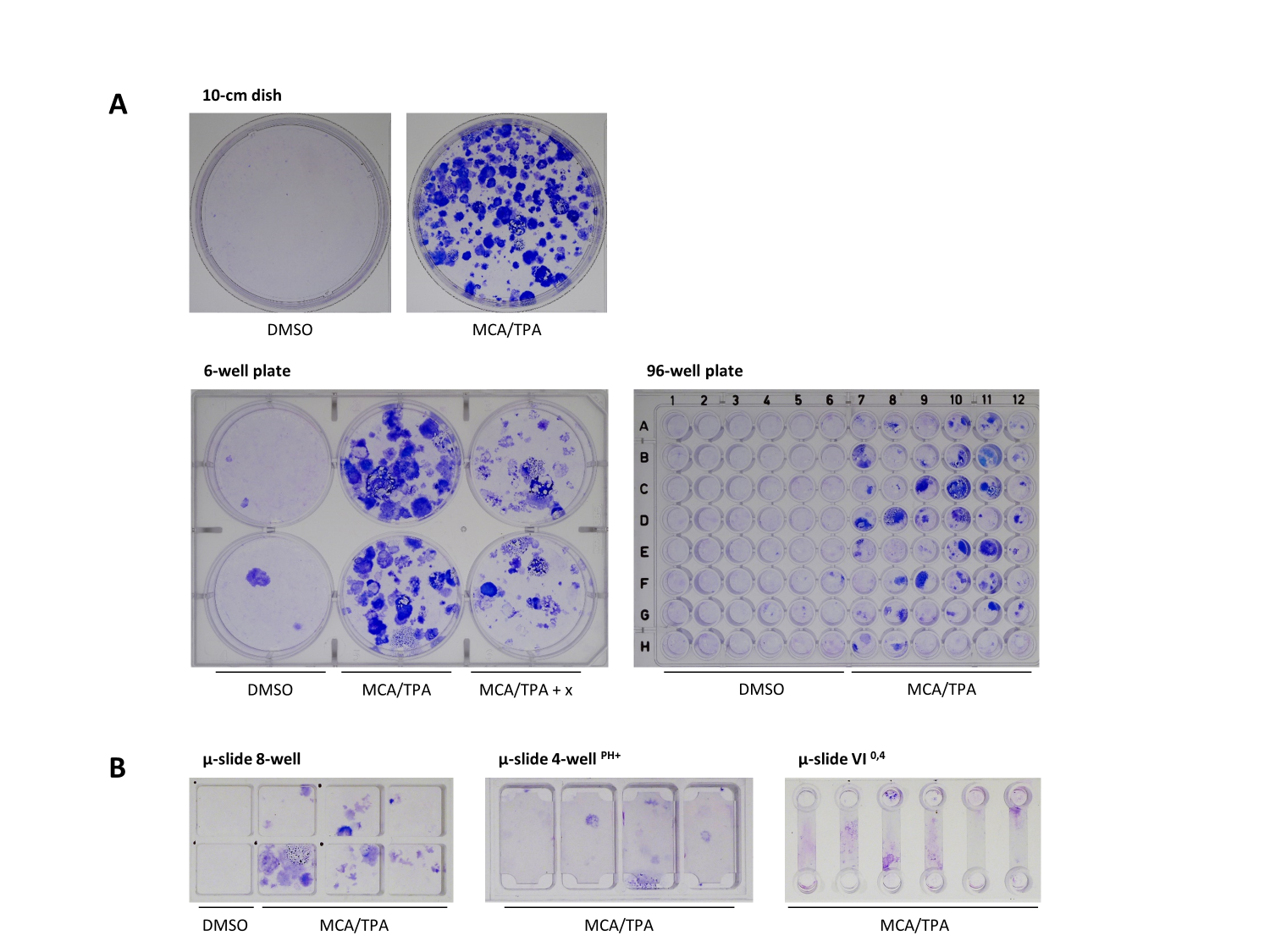
**

Transfer of the BALB/c assay in different plate and dish formats. (A) The standard BALB-CTA is performed in 6-well plates with DMSO as negative control, MCA/TPA as positive control and can be extended by the parallel incubation with other substances (displayed as + x). For other applications it is also possible to use the wide range of well plates or culture dishes to investigate cell transformation. The 96-well plate is quiet useful to test several substances in the BALB/c cell transformation assay in a high-throughput manner. Furthermore the BALB/c cell transformation protocol can be performed in larger cell culture dishes (shown for 10-cm dish) to achieve high amounts of protein for immunoblot or cells for oxygen measurements. (B) For immunofluorescence and high-end microscopy the different µ-slides form ibidi (8-well µ-slide; 4-well µ-slide with a special intermediate plate for excellent phase contrast or the 6-channel slide for flow experiments) are quiet beneficial. Foci forming potential with MCA/TPA incubations in all plate formats is visualized by giemsa staining (blue colored).
